# Overcoming Methicillin-Resistance *Staphylococcus aureus* (MRSA) Using Antimicrobial Peptides-Silver Nanoparticles

**DOI:** 10.3390/antibiotics11070951

**Published:** 2022-07-15

**Authors:** Mohammad Asyraf Adhwa Masimen, Noor Aniza Harun, M. Maulidiani, Wan Iryani Wan Ismail

**Affiliations:** 1 Cell Signalling and Biotechnology Research Group (CeSBTech), Faculty of Science and Marine Environment, Universiti Malaysia Terengganu, Kuala Nerus 21030, Terengganu, Malaysia; asyrafadhwa@gmail.com; 2 Advanced NanoMaterials (ANOMA) Research Group, Faculty of Science and Marine Environment, Universiti Malaysia Terengganu, Kuala Nerus 21030, Terengganu, Malaysia; nooraniza@umt.edu.my; 3 Faculty of Science and Marine Environment, Universiti Malaysia Terengganu, Kuala Nerus 21030, Terengganu, Malaysia; maulidiani@umt.edu.my; 4 Biological Security and Sustainability Research Group (BIOSES), Faculty of Science and Marine Environment, Universiti Malaysia Terengganu, Kuala Nerus 21030, Terengganu, Malaysia

**Keywords:** antibiotic resistance, antimicrobial peptides, MRSA, silver nanoparticles

## Abstract

Antibiotics are regarded as a miracle in the medical field as it prevents disease caused by pathogenic bacteria. Since the discovery of penicillin, antibiotics have become the foundation for modern medical discoveries. However, bacteria soon became resistant to antibiotics, which puts a burden on the healthcare system. Methicillin-resistant *Staphylococcus aureus* (MRSA) has become one of the most prominent antibiotic-resistant bacteria in the world since 1961. MRSA primarily developed resistance to beta-lactamases antibiotics and can be easily spread in the healthcare system. Thus, alternatives to combat MRSA are urgently required. Antimicrobial peptides (AMPs), an innate host immune agent and silver nanoparticles (AgNPs), are gaining interest as alternative treatments against MRSA. Both agents have broad-spectrum properties which are suitable candidates for controlling MRSA. Although both agents can exhibit antimicrobial effects independently, the combination of both can be synergistic and complementary to each other to exhibit stronger antimicrobial activity. The combination of AMPs and AgNPs also reduces their own weaknesses as their own, which can be developed as a potential agent to combat antibiotic resistance especially towards MRSA. Thus, this review aims to discuss the potential of antimicrobial peptides and silver nanoparticles towards controlling MRSA pathogen growth.

## 1. Introduction

Antibiotics are one of the outstanding discoveries in the medical field in treating infectious diseases caused by pathogenic bacteria. Before the antibiotic discovery era, the lethality and death rate caused by pathogenic microorganisms was high until the accidental rediscovery of penicillin in 1928 by Alexander Fleming [1]. This rediscovery grants the exploration of other types of antibiotics such as sulphonamides, lipopeptides, aminoglycosides, fluoroquinolones, and many more [1,2]. Antibiotics also allow modern medical technology to exist as it aids in preventing infection in chemotherapy and various surgical wounds. 

Although antibiotics give significant advantages in treating diseases caused by pathogenic bacteria, Alexander Fleming warns of the danger of uncontrolled antibiotic usage where resistance can be developed. The warning appeared to be true as *Escherichia coli* started to exhibit antibiotic resistance (AR) towards penicillin in 1940 [3]. Up until this day, antibiotic resistance has been a significant threat in the healthcare system as more bacteria developed resistance towards various classes of antibiotics. It is predicted that, by 2050, AR related death may reach 10 million per year [4,5].

A recent comprehensive report released in The Lancet [6] stated that 4.95 million AR associated death and 1.27 million AR attributed death were estimated from 204 countries in 2019. Highest AR related death can be found in Western Sub-Saharan Africa with estimated 27.3 AR attributed death per 100,000 and 114.8 AR associated death per 100,000. Meanwhile, the lowest death can be found in Australasia where only 6.5 AR attributed deaths per 100,000 and 28 AR associated deaths per 100,000. The same report also lists out six pathogenic bacteria that cause the most death in 2019 [6]. In order of the number of deaths, *E. coli*, *S. aureus*, *K. pneumoniae*, *A. baumannii*, and *P. aeruginosa* caused 929,000 AR attributed deaths and 3.57 million AR associated deaths.

Methicillin-resistant *Staphylococcus aureus* (MRSA) is an antibiotic-resistant type of *S. aureus* that is generally resistant towards beta-lactam antibiotics such as penicillin (methicillin and oxacillin) and cephalosporin [7,8,9]. Beta-lactam inhibits the bacterial growth by halting the cell wall synthesis process [10,11,12]. MRSA generally overcomes the beta-lactam effects by producing beta-lactamase and altering the binding site for cell wall synthesis [7,8,9,13]. The current clinically approved method to treat MRSA infection involves different antibiotic classes such as vancomycin and teicoplanin [14,15]. These glycopeptide antibiotics act on the bacterial cell wall similar to beta-lactam, but it utilises different target by binding to the peptidoglycan side chain, which prevents peptidoglycan crosslinking [13,14,15]. However, the newer MRSA strain started to exhibit resistance towards glycopeptide antibiotics, which makes it difficult to treat the infection [13,14]. Other types of antibiotics such as mupirocin, clindamycin, fusidic acid, and co-trimoxazole also used a second line option in treating MRSA [16]. However, these antibiotics can only be prescribed when there is no other alternative available due to the risk of resistance [16,17]. Thus, alternatives to treat MRSA without the use of different classes of antibiotics are greatly needed. 

Recent scientific development showed some promising potential in inhibiting MRSA through the usage of antimicrobial peptides (AMPs) and silver nanoparticles (AgNPs). These two agents exhibit broad-spectrum antimicrobial properties, which makes them the suitable candidates to combat MRSA threat [18,19,20,21]. AMPs are naturally occurring molecules that can be found in all types of life, which are involved in innate immunity defense [20,21]. AMP mainly takes action on the bacterial membrane, and it can be simplified into two mechanisms of action: membranolytic and non-membranolytic action [21,22,23]. Membranolytic action can be defined as direct AMP action on the bacterial membrane, which greatly alters its structural integrity [23,24,25]. Meanwhile, non-membranolytic action is when AMPs were internalised into the cells without causing major damage to the membrane, but it targets the vital intracellular components [26,27,28].

AgNPs are metallic nanoparticles that have unique physicochemical properties including optical, thermal, electrical and high electrical conductivity in comparison to its bulk form due to its nano size [29,30]. Their enhanced antimicrobial properties mainly contributed with their large surface area per volume area, which allows more antibacterial contact with the pathogenic bacteria [19,31,32]. AgNPs are also steadily gaining interest due to its multiple mechanism of actions. AgNPs generally act on membranes by disrupting it through hole formation, direct adhesion and internalisation into the membrane, excessive ROS generation and alteration in signalling pathways [30,33,34,35]. Despite their excellent antimicrobial properties, AMPs and AgNPs have their own limitations, but, through the combination of both agents, a positive synergistic effect can be observed [36,37]. Thus, this review discusses about MRSA, its mechanism of resistance, advantages and limitations of AMPs and AgNPs as its own. This topical review also discusses the combination of AMPs–AgNPs in combating bacteria, particularly MRSA and *S. aureus*, as no other review has been reported with the combination of the two antimicrobial agents. The review process was done based on a literature search in Google Scholar with the keywords, antimicrobial peptide, antibiotic resistance, MRSA, silver nanoparticles and *S. aureus*, with most of it published from 2016–2022 and some of the papers published before 2016.

## 2. Methicillin-Resistant *Staphylococcus aureus*

*Staphylococcus aureus* is Gram-positive bacteria with round shape morphology that commonly can be found in the body as a part of its microbiota. Despite it acting commensally on the human body, it can be opportunistic bacteria since it can cause skin infections and food poisoning. Methicillin-resistant *Staphylococcus aureus* (MRSA) is an antibiotic-resistant strain of *S. aureus* that are mainly resistant to beta-lactam antibiotics. MRSA was first identified in 1961 in United Kingdom just a year after methicillin was introduced to treat *S. aureus* infection [38,39]. Despite methicillin no longer being used clinically, the term methicillin-resistant is still used to reflect *S. aureus* resistance towards commercial antibiotics such as beta-lactams antibiotics including oxacillin. According to World Health Organization (WHO) and the Centers for Disease Control and Prevention (CDC), MRSA has been a big and serious threat on the pathogenic bacteria watch list respectively [40,41]. According to recent systematic analysis in the Lancet in 2019, MRSA alone caused more than 100,000 deaths [6]. Originally, MRSA are common in the healthcare setting, and this type of MRSA is often dubbed as healthcare-associated or hospital-acquired MRSA (HA-MRSA) [42]. The infection can be spread through direct contact with an infected wound or contaminated hands. Untreated infection can cause serious bloodstream infections, surgical site infections, sepsis and pneumonia [7,43]. Other types of MRSA are community-associated (CA-MRSA) and livestock-associated MRSA (LA-MRSA) [39,43].

Beta-lactam antibiotics act on the bacterial cell wall by binding to the penicillin binding protein (PBP), which is responsible for the crosslinking of N-acetylmuramic acid (MurNAc) and N-acetylglucosamine (GlcNAc) [10,11]. This crosslinking will form a cell wall that protects the bacteria from external threats. MurNAc subunits have pentapeptide chains attached to it, typically with a sequence of l-Ala-γ-d-Glu-l-lysine (or -meso-diaminopimelic acid)-d-Ala-d-Ala [11]. Beta-lactam antibiotics such as penicillin, cephalosporin, carbapenem and monobactams have a beta-lactam ring which shared similar structural homology to d-Ala-d-Ala of the pentapeptide chain [10,44]. The d-Ala-d-Ala substrate is responsible for the PBP binding site for crosslinking, and this similarity causes beta-lactam antibiotics bind to PBP, causing the crosslinking between the glycan stands to be halted [11]. The binding between beta-lactam and PBP causes the build-up of peptidoglycan precursors which trigger autolytic digestion of old peptidoglycan by hydrolase [10]. Without the production of new peptidoglycan, the structural integrity of the cell wall is significantly disrupted and led to cell damage due to high internal osmotic pressure [11,12].

**Figure 1:**
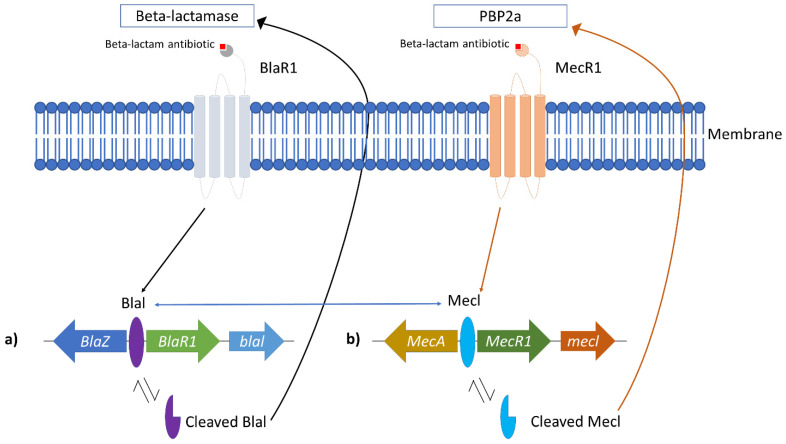
Correlation between Blal and MecA role in MRSA resistance. (**a**) *bla* operon responsible for beta-lactamase production and (**b**) *mec* operon responsible for the alteration of normal PBP to PBP2a. The blue arrows indicate that *bla* and *mec* operon shared similarities, which allows the repressor (Blal and Mecl) to bind to each operon.

MRSA overcomes this detrimental effect by producing beta-lactamase, an enzyme to break down the antibacterial effect of beta-lactam antibiotics and production of the *mecA* gene, which changes the penicillin-binding protein (PBP) confirmation. Beta-lactamase is an enzyme produced by bacteria to counteract the effects of beta-lactam antibiotics. This enzyme hydrolyses beta-lactam in the periplasmic space, thus deactivating it before PBP interaction [4]. Beta-lactamase production in staphylococci is controlled by the repressor BlaI and the sensor protein BlaR1 (Figure 1a) [44]. The genes encoding beta-lactamase, the *blaZ-blaR1-blaI* genes, are repressed by BlaI is from transcribing beta-lactamase when beta-lactam is absent [10,12]. Once beta-lactam is presented, the transmembrane sensor, BlaR1, covalently binds to it and irreversibly acylated at its active site serine. This will activate the intracellular zinc metalloprotease domain of BlaR1 and cause BlaI that are bound to *blaI-blaRI* operator to proteolytically cleave and dissociate from its binding site [12]. The dissociation allows *blaZ* to be upregulated and transcribed beta-lactamase enzyme. The produced beta-lactamase enzyme later hydrolyses beta-lactam antibiotic by hindering it from binding with PBP [10,12]. Thus, the peptidoglycan synthesis of the bacteria can be initiated as usual.

In MRSA, the PBP responsible for the peptidoglycan cross-linking is altered to novel penicillin-binding protein 2a (PBP2a), which has a lower binding affinity to beta-lactam antibiotics [39]. The resistance arose from the *mecA* gene located in the staphylococcal cassette chromosome *mec* (*SCCmec*), and this resistance gene can be passed to other populations through horizontal gene transfer [12]. Upon acquiring the *mecA* gene, it will be localized in the *S. aureus* chromosome. The production of PBP2a is controlled by MecI repressor and transmembrane MecR1 sensor protein (Figure 1b) [10]. In the absence of beta-lactam antibiotics, MecI represses *mecA* gene expression by binding to the promoter region of *mec* operon [10,39]. In the presence of beta-lactam antibiotics, the antibiotic binds to the MecR1 sensor protein. It triggers autolytic activation of the metalloproteinase domain in the cytoplasm part of MecR1, causing signal transduction to be activated [12]. The latter caused the MecI repressor to be proteolytically cleaved from its binding site, and this allows the expression *mecA* to produce PBP2a [10]. The PBP2a production allows the peptidoglycan wall synthesis to continue without the interaction of beta-lactam antibiotics due to its low binding affinity to the antibiotic [7,39]. Interestingly, the *mec* operon shared a similar structure and function with the *bla* operon, which produces beta-lactamase [7,12]. This similarity allows the Blal repressor to bind to the *mec* operon to repress *mecA* transcription (Figure 1) [10].

## 3. Antimicrobial Peptides (AMPs)

**Figure 2:**
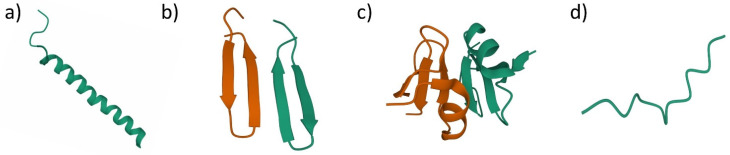
Antimicrobial peptides structural classification based on their secondary structure. (**a**) alpha-helices AMPs, human LL-37 (PDB ID: 2K60); (**b**) beta-sheets AMPs, protegrin-1 (PDB ID: 1ZY6); (**c**) mixed structure AMPs, human beta-defensin-2 (PDB ID: 1FD4); (**d**) extended structure AMPs, indolicidin (PDB ID: 5ZVN).

Antimicrobial peptides (AMPs) are naturally occurring host defense mechanisms against infections. AMPs can be found in all living organisms such as plants, microorganisms and animals [20,23]. Typical AMPs consist of 5–50 amino acid chains and have amphipathic or cationic structure. Despite AMPs being naturally occurring, synthetic AMPs have been developed by the scientist to overcome the naturally occurring AMP limitations [21]. While naturally occurring AMPs are susceptible to proteolytic degradation, synthetic AMPs have a longer half-life, and it is designed to improve their antimicrobial properties. AMPs then can be divided into four main groups based on its secondary structure including amphipathic alpha-helices, beta-sheets, a combination of both alpha and beta structure (mixed) and extended structure (without alpha and beta structure) (Figure 2) [21]. 

Alpha helices’ AMPs generally contain two amino acids that are adjacent to each other with the distance of 1.5 Å (0.15 nm) [23]. The most studied AMPs in this group is LL-37 (amino acid sequence: LLGDFFRKSKEKIGKEFKRIVQRIKDFLRNLVPRTES), AMPs that can be found in the human body that act as the host defense towards bacterial infections [21,23,45,46]. Beta-sheets’ AMPs have at least two beta strands with disulfide bonds. Protegrin-1 (amino acid sequence: RGGRLCYCRRRFCVCVGR-NH_2_), which is isolated from pigs, is one of the examples in this group which exhibits antimicrobial activity against fungi, bacteria and some enveloped viruses [23,26]. Mixed structure usually consists of a combination of alpha-helix and beta-sheet that are packed against each other. Human-beta-defensin-2 is one of the well-studied AMPs in this group [23,28]. The extended structure is a unique group of AMPs that consists of two or more tryptophan, arginine, histidine and proline structure in single molecules. Cattle neutrophil isolated AMPs, indolicidin (amino acid sequence: ILPWKWPWWPWRR-NH_2_), are in this group [23,45]. 

**Figure 3:**
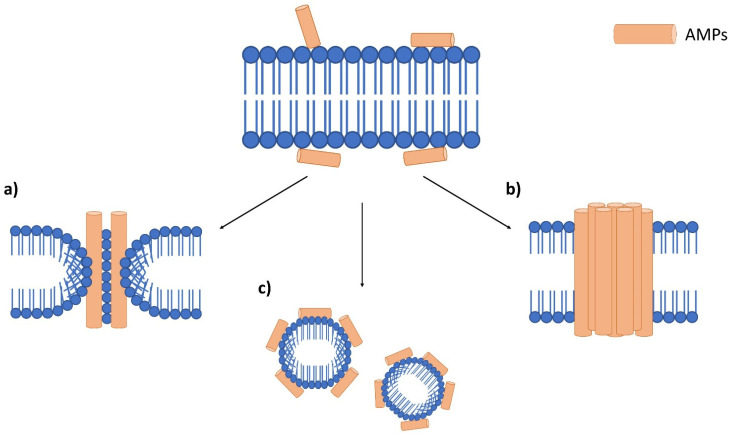
Antimicrobial peptide mechanism on the bacterial membrane. Accumulation of AMPs on the bacterial membrane surface, which leads to three main membranolytic mechanisms. (**a**) toroidal pore model which forms pores on the membrane, (**b**) barrel-stove model which AMPs aggregate before entering the membrane and (**c**) a carpet-like model which promotes the formation of micelles.

In terms of AMP mechanism of actions, it can be divided into two main categories, membrane disruptive and non-membrane disruptive AMPs [22,26,28]. Membrane disruptive AMP can be further divided into the toroidal-pore model, barrel-stave model and carpet model (Figure 3) [26,27]. The toroidal-pore model is where AMPs form pores in the membrane (1–2 nm diameter) vertically [27]. This will also cause the phospholipid head to bend due to the insertion of AMPs [28]. In the barrel-stave model, AMPs bind to the cell membrane and aggregate before penetrating the membrane [26]. During this process, hydrophobic regions of AMPs are inserted into the phospholipid membrane, while the hydrophilic regions of AMPs are facing the outer part of the membrane pore [27]. This will cause uncontrolled cellular movement for the cell, which will lead to cell death. The carpet-like model destroys the membrane in a detergent-like manner [22]. AMPs are first arranged onto the cell membrane by their hydrophobic part facing the phospholipid bilayer, which alters its surface tension. The altered surface tension later causes micelles formation as the results of peptide accumulation and destroys the membrane [22,27]. 

A non-membrane disruptive mechanism is rarely studied in AMP research, but recent advancement showed that AMPs are internalised into cells and interacts with vital intracellular targets and even inhibits cell wall biosynthesis [28]. This includes inhibition of protein and nucleic acid synthesis, cell division and protease activity [23,26,28]. AMPs inhibit protein synthesis by directly interacting with the transcription and translation process. PR-39 AMPs isolated from a pig’s small intestine can inhibit protein synthesis, which causes proteins degradation that are required for DNA synthesis [28]. Indolicilin induces degradation of nucleic acid by binding to the double stranded DNA, which causes the DNA synthesis to be halted [23,26]. Teixobactin AMPs bind to lipid II and lipid III (precursors of cell wall), which later inhibits the cell wall synthesis process [28]. Based on the promising antimicrobial action of AMPs, Table 1 showed some examples of AMPs that are effective towards MRSA and wild type *S. aureus*.

As the innate immune system, AMPs have broad spectrum antimicrobial properties which are said to be effective towards pathogenic microorganisms [21,23,45]. These antimicrobial properties are greatly enhanced as AMPs can be found abundantly at the site of the infection, which makes it more time efficient since it can react faster to combat the infection [23,28]. Resistance towards AMPs is also said to be low, which makes it one of the suitable candidates to combat MRSA [28]. Besides that, AMPs also have good water solubility and thermal stability [23,45]. However, AMPs do possess weakness as naturally occurring AMPs are susceptible towards proteolytic degradation, which limits their potential [23,28]. In addition, AMP production and purification can be costly sometimes. Despite their broad antimicrobial spectrum, it can be a challenge to be used medically as some AMPs might induce hypersensitivity after application and might cause immunogenicity and toxicity when it is administered in humans [21,23,45].

## 4. Silver Nanoparticles (AgNPs)

**Figure 4:**
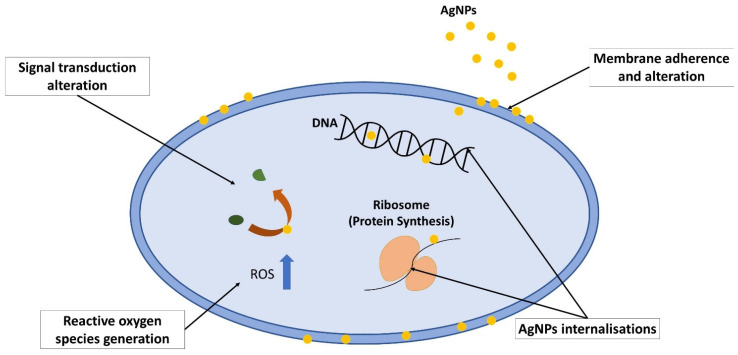
Mechanism of action exhibited by silver nanoparticles on bacteria, which includes membrane adherence and alteration, AgNP internalisations, which later induces cellular damage, reactive oxygen species generation, which causes oxidative stress, and signal transduction alteration.

Silver nanoparticles are the product of nanotechnology which are particles of silver that are ranging in size from 1 to 100 nm [38,39]. Their nanosized greatly enhances its broad-spectrum antibacterial properties as it has larger surface area per volume ratio [29,33,35,51]. Due to their unique properties in terms of optical, electrical, magnetic and antibacterial, AgNPs have various applications, which include medical appliances, optical sensors, cosmetics, drug delivery, textiles, keyboards, wound dressings and food packaging [35,51,52,53]. AgNPs are gaining popularity due to their multiple mechanisms of action on bacteria (Figure 4), which include direct adhesion of AgNPs on the bacterial surface and altering the membrane structural integrity [32,54]. Next, AgNPs penetrate inside the bacterial cell and interact with its intracellular components, damaging it until it cannot perform vital cellular processes [32,54]. AgNPs are also able to induce reaction oxygen species and free radical generation, thus causing irreversible oxidative damage to the bacteria [55,56]. Alteration of vital signaling transduction, which is necessary for the bacterial life cycle, is also one of the mechanisms exhibited by AgNPs [52,57].

When AgNPs is in contact with the outer membrane of the bacteria, it adheres to it due to the difference in electrostatic charge [58]. This electrostatic force is driven by the positively charged AgNPs and negatively charged bacterial cell membrane [34,59]. The negative charge on the membrane is contributed by the presence of the amino, carboxyl and phosphate group [60,61]. This metal depletion on the membrane causes pit formation on the membrane as first visualized using transmission electron microscopy by Sondi and Salopek-Sondi [62]. The phenomenon causes the membrane to fail to regulate vital cellular content movements and may lead to cell death.

After AgNPs attached on the membrane, its permeability and structural integrity are greatly altered, and this causes some portion of AgNPs to infuse into the cell. This statement is further strengthened by several studies which revealed that AgNPs penetrate the cells through transmission electron microscope analysis on bacteria [19,62,63]. In addition, in the presence of oxygen and proton, AgNPs dissociate to Ag^+^ ions, which also facilitates the infusion [64]. When AgNPs are internalised into the cell, it interacts with cellular molecules and structures such as protein, DNA and lipids. For instance, AgNPs interrupt protein synthesis by interacting with ribosome by denaturing it, which halts the translation process [65,66]. AgNPs also interacts with DNA molecules, which may cause denaturation and shearing of DNA and also cell division interruption [32,67]. The interaction causes the bacteria to lose the ability to undergo division, reproduction and eventually cell death [54,68]. 

Reactive oxygen species or ROS are also the culprits for the bacterial growth inhibition. AgNPs generate a high level of ROS, which induces oxidative stress in the cell [55,56]. Oxidative stress causes a vital cellular component breakdown, such as protein, RNA and DNA, which led to the alteration of membrane permeability and increased cellular component leakage from the cell [52,69]. This will cause irreversible oxidative damage to the bacteria and cell death [33,65].

Tyrosine phosphorylation is important in activation of various proteins such as RNA polymerase sigma factor that are essential in the bacterial transcription process [34,57]. Increased dephosphorylation of tyrosine profile might inhibit vital processes in bacteria such as polysaccharide biosynthesis of bacterial capsule in bacteria [33,34]. AgNPs can alter tyrosine phosphorylation, which led to failed regulation of the cellular process and homeostasis, which later destroys the cells [52,57]. Since AgNPs managed to exhibit multiple mechanisms of action on bacteria, it is said to be effective to combat MRSA. Table 2 showed some AgNPs action on inhibiting the growth of MRSA.

Despite AgNPs possessing broad spectrum antibacterial activity on MRSA, it tends to aggregate and reduces its antibacterial properties [36,77]. Other than that, some AgNPs can be toxic in vitro and in vivo when it is administered as its own without a capping agent, which limits its toxicity effect towards cells [78]. Oxidation of AgNPs also contributes to its weakness to be developed as a promising antibacterial agent [78,79,80].

## 5. AMP and AgNPs Combination on MRSA or MSSA

Despite AMPs and AgNPs having their own weaknesses on their own, the combination of these two, or sometimes with the addition of polymer, enhances its antibacterial properties while greatly reducing their toxicity effects. Synergistic effect in terms of stronger antibacterial activity of these two agents can also be observed once they are administered together.

A study by Jin et al. utlises AMPs, Tet-213 and AgNPs that are loaded onto porous silicon microparticles [36]. Tet-213 is a 10 amino acid peptide (sequence: KRWWKWWRRC) that possesses broad spectrum activity due to the presence of thiol group and, with the combination of AgNPs, the antimicrobial effect increases drastically. The presence of porous silicon microparticles (PSiMPs) acts as a carrier for effective delivery of the antimicrobial agent to the infected site [36,81]. PSiMPs was chosen due to its tunable pore size, biocompatibility and decompatibility. However, PSiMPs only dissociate in an alkaline condition as it is normally acidic during the early stage of infection [82]. Despite the carrier only being able to dissociate in alkaline conditions, the presence of ROS also allows PSiMPs be to be dissociated easily. When ROS is high during the wound infection, it allows the carrier to be disintegrated and releases silver ions from AgNPs together with Tet-213. The acidic condition also allows a gradual release of AgNPs-AMPs, which allows more effective and stable antimicrobial action. In this study, for the combination of these agents, the MIC value was greatly reduced to 2 mg/mL in comparison to AgNPs-PsiMPs (2.5 mg/mL) and AMPs-PsiMPs (>5 mg/mL) on *S. aureus* [36]. In-vitro testing on mouse fibroblast (NIH3T3) cells and human immortal keratinocyte (HaCaT) showed low toxicity effects as this complex does not affect the cells’ proliferation. This AgNPs-AMPs-PSiMPs combination also exhibits low toxicity and faster wound healing on rats infected with *S. aureus* [36]. The faster wound healing contributed with the release of silicon ions in the complex, with the help of AgNPs and AMPs to reduce the bacterial infection in the wound. Note that silicon ions promote wound healing by activating the epidermal growth factor receptor (EGFR), epidermal growth factor (EGF) and extracellular signal-related kinase (ERK) signaling pathway [36,83,84].

A star conjugated PCL-*b*-AMPs nanocomposite was also used in stabilising AgNPs and enhancing antimicrobial activity of it with the help of AMPs [77]. Star conjugated PCL-*b*-AMPs consist of polycaprolactone (PCL) and polypeptide (Phe_8_-*stat*-Lys_32_), which are later loaded with AgNPs. This complex is relatively stable at room temperature for three months with any sign of aggregations. In this case, PCL-*b*-AMPs penetrate the negatively charged membrane since this complex is positively charged. This penetration allows AgNPs to be released in the cytoplasm and the deactivating of vital cellular components. This complex managed to exhibit enhanced inhibition on *S. aureus* (27.6 mm) when compared to the combination of PCL-*b*-AMPs (19.1 mm) and AgNPs (12.7 mm) alone. A low MIC value (4 µg/mL) is also observed when PCL-*b*-AMPs with AgNPs is tested on MRSA [77]. This suggests that a synergistic effect of AMPs and AgNPs allows higher inhibition on the bacterial growth. A damaged membrane was also observed on MRSA, which later led to cell death [77,85]. This complex also showed no sign of resistance even after 21 passage exposure with a sub-lethal MIC value of the complex when tested on the wild type *S. aureus* [77]. It also showed low cytotoxicity towards normal mouse fibroblast cells (L929) as it managed to retain up to 80% of cell viability after 48 h. The PCL-*b*-AMPs managed to reduce AgNPs toxicity by only releasing it to the target site besides from their biocompatibility.

Polymersomes, which are polymeric biocompatible vesicle, were also used for an effective synergistic antimicrobial effect of AMPs and AgNPs [85]. PR-39 peptide was utilised in the polymeric compound as it is effective towards inhibiting bacterial growth. Originally, porcine PR-39 peptide could not translocate across the bacterial membrane as MRSA produces protease which degrades the AMPs. For the addition of polymersomes and AgNPs, the MRSA growth was totally eradicated under 23 h [85]. Polymersomes and AgNPs allow the complex to translocate the cells and release the antimicrobial agent to inhibit the bacterial growth. From the scanning electron microscopy, apparent damage on MRSA membrane can be observed, which led to cell death [27,85]. A low toxicity level toward CCL-110 human dermal fibroblast (HDF) cell lines can be observed since the coating reduces the toxicity effects of AgNPs and stabilises AMPs [77,85].

A combination of protegrin-1 AMPs and gelatinized coated AgNPs also greatly enhances its antimicrobial properties as it exhibits low MIC value (6 µg/mL) in comparison to AgNPs (48 µg/mL) and AMPs (8.5 µg/mL) treatment alone [79]. It is said that this complex limits MRSA growth by membrane permeabilisation (possibly through the toroidal pores model) [28,79]. The same study also combines AgNPs with another type of AMPs, Indolicidin [79]. This combination also exhibits excellent antimicrobial properties as its MIC value to inhibit MRSA is 12 µg/mL. The MIC value for indolicidin alone on MRSA 40 µg/mL is relatively high in comparison to the AgNPs-Indolicidin complex. This complex acted on MRSA by self-translocating into the cells by forming an apparent pore on the membrane and interacting with nucleic acid, which halts the DNA synthesis [22,27]. Low haemolytic activity can be observed when the complex was tested with human erythrocytes. However, more optimisations are required as they showed a cytotoxicity effect towards cancerous and normal cell lines, which grants in vivo assessment to elucidate the actual toxicity.

A novel composite of AgNPs and designed AMPs P-13 (amino acid sequence: KRWWKWWRRCECG) were tested against *S. aureus* (ATCC 25923) [86]. Based on the MIC values, this composite manages to inhibit bacteria effectively at lower concentration (7.8 ± 0.05 µg/mL) compared to AgNPs and AMPs alone with 7.8 ± 0.05 µg/mL and >500 ± 0.04 µg/mL, respectively. Interestingly, with the addition of P-13 to AgNPs, a drastic toxicity reduction can be observed on mouse fibroblast cells (NIH-3T3) [86]. This addition allows AMPs to stabilise AgNPs and reduce its cytotoxicity effect in comparison to AgNPs alone. It is proposed that this complex inhibits bacteria growth by adhesion to the bacteria through electrostatic force and was internalised into the cell reacting with vital cellular components. This causes cellular leakage out of the cell, which led to cell death [27,86].

Another study by Li et al. developed multifunctional peptide (MFP)-coated silver nanoparticles as an alternative to combat antibiotic resistance [78]. In this study, AMPs tachyplesin-1 and target peptide N-ac-PGP-PEG were combined to adsorb AgNPs through electrostatic interaction. This complex was proven to be effective at inhibiting *S. aureus* and MRSA growth with MIC values of 8 µg/mL and 16 µg/mL, respectively [78]. Despite the MIC for vancomycin, an antibiotic control in this experiment is much lower than the complex (2 µg/mL); this complex was proven to be a promising agent to inhibit the bacterial growth with future optimisations.

The AMP@PDA@AgNPs nanocomposite was created through polymerisation to inhibit biofilm formation by *S. aureus* [80]. PDA was added as a delivery agent, which allows more effective AMPs and AgNPs delivery to the target site. This allows more effective internalisation into the cell to exhibit its antimicrobial activity. This nanocomposite showed no cytotoxicity effect even at a high concentration (400 µg/mL) when tested on human embryonic kidney (HEK293T) cells. To inhibit *S. aureus* growth, only a concentration of 25 μg/mL was required, which is much lower than the concentration used in the cytotoxicity assessments. This complex also managed to reduce biofilm formed by the bacteria by reducing the expression of biofilm forming genes (las I and rh II, fim H) [80]. Table 3 showed other combinations of AMPs with AgNPs that are able to inhibit *S. aureus* or MRSA growth effectively.

**Figure 5:**
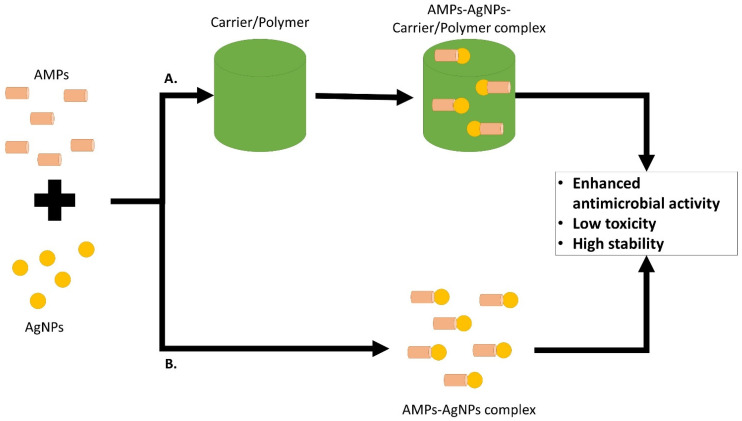
Combination of AMPs and AgNPs or together with the addition of carrier/polymer for a more effective delivery system to the target site, enhanced antimicrobial activity and lower toxicity effect in comparison to the agents on their own.

In general, AMPs and AgNPs can be combined together to exhibit synergistic antimicrobial effect or carrier/polymer can be added to the complex to allow more effective AMPs and AgNPs for the target site without exhibiting a high toxicity effect (Figure 5).

## 6. Conclusions and Future Perspectives

Despite as antibiotic resistance threat that keeps on increasing year by year, scientists are never giving up on finding alternatives to curb the spreading of antibiotic resistance. The emergence of antimicrobial peptides with multiple membranolytic and non-membranolytic mechanism cast light on the antibiotic resistance research. A lower rate of microbial resistance towards AMPs also allows it to be studied intensively in combating MRSA, a worldwide pathogenic threat. The introduction of silver nanoparticles in this modern era also allows its utilisation in combating antibiotic-resistant bacteria including MRSA. Multiple mechanisms, such as direct adherence and internalization of AgNPs, allow it to exhibit an antimicrobial effect effectively by inducing ROS and alteration of signal transduction. Nevertheless, AMPs and AgNPs each possess their own weaknesses, which include toxicity and instability. A combination of these two agents somehow overcomes these weaknesses by stabilising these agents to the target site. A synergistic effect can also be observed once these two agents are combined to inhibit bacterial growth. A lower toxicity effect in vitro and in vivo can also be observed. Although this research is still the beginning, future optimisations can be done, especially in terms of enhanced complex stability, lower dosage required in inhibiting bacteria and lower toxicity, in vitro and in vivo need to be done. Clinical research also needs to be done before the co-application of AMPs and AgNPs can be truly used in combating antibiotic resistance, especially towards MRSA in humans.

## Figures and Tables

**Table 1:** Examples of natural antimicrobial peptides that are effective towards *Staphylococcus aureus* (methicillin-susceptible and MRSA).

AMPs Type(s)	AMPs Origin/Type	Amino Acid Sequence	Antibacterial Activity on *Staphylococcus aureus*	Ref.
Temporin A	Isolated from frog skin secretion, *Rana temporaria*	FLPLIGRVLSGIL-NH2	Effective toward methicillin-susceptible *S. aureus* (MSSA) and MRSA. Exhibit MIC value of 4 µg/mL once tested on surgical wound isolated MRSA.	[18]
			Exhibit MIC values of 16–64 µg/mL once tested on 215 isolates of MSSA and MRSA	[47]
Cecropin A-melittin hybrid peptide [CA(1–7)M(2–9)NH2]	Hybrid peptide derived from cecropin A and melittin partial sequence	KWKLFKKIGAVLKVL-NH2	Effective towards MRSA. Exhibit MIC value of 8 µg/mL once tested on skin lesion isolated MRSA.	[18]
			Exhibit MIC values of 4 mg/mL to 32 mg/mL once tested on 215 isolates of MSSA and MRSA	[47]
Citropin 1.1	Isolated from frog’s dorsal and submental glands *Litoria citropa*	GLFDVIKKVASVIGGL-NH (2)	Exhibit MIC value of 16–64 mg/mL once tested on 215 isolates of MSSA and MRSA	[47]
			Effective towards MRSA. Exhibit MIC value of 16 µg/mL once tested on wound, deep wound and skin lesion isolated MRSA.	[18]
			Exhibit MIC value of 32 µg/mL once tested on MRSA strain JE2	[48]
Cathelicidin LL-37	Human derived cathelicidin AMPs	LLGDFFRKSKEKIGKEFKRIVQRIKDFLRNLVPRTES	Effectively reduces infection once tested on MRSA infected wound on mice in comparison to the antibiotic groups (teicoplanin).	[49]
			Exhibit MIC values on biofilm forming MSSA and MRSA (isolated from chronic wound) 89.6 mg/L and 132.3 mg/L, respectively. Inhibit the growth by affecting quorum sensing and biofilm gene expression.	[50]

**Table 2:** Antibacterial effect of AgNPs on MRSA.

Silver Nanoparticles’ Properties	Antibacterial Action	Ref.
Spherical shape with the size ranges from 8.55 to 20.3 nm	Exhibit MIC value 8.125 µg/mL on MRSA. It said the AgNPs inhibit MRSA by adhering and penetrating the cell by interacting with vital cellular compounds.	[70]
Spherical AgNPs with the size range from 5–10 nm	Exhibit MIC value ranging from 11.25 µg/mL to 45 µg/mL on MRSA. AgNPs disrupt the biofilm formed by MRSA once visualised using a scanning electron microscope.	[71]
Spherical AgNPs with the size 150 nm that are determined by dynamic light scattering	Showed inhibition on disk diffusion assay and exhibited MIC value at 0.015 mg/mL on all tested MRSA strains.	[72]
Spherical AgNPs with the size range <100 nm (Three different AgNP sizes used in the experiment. AgNPs 1:36 nm, AgNPs 2:113 nm and AgNPs 3:78 nm)	Smaller AgNPs (AgNPs 1:36 nm) showed higher MRSA inhibition due to higher AgNP contact rate with bacteria based on a disk diffusion assay. MIC value of MRSA upon interaction with AgNPs is 0.50 μg/mL.	[73]
Spherical AgNPs with diameter of 9 nm	Exhibit MIC value of 1.95 µg/mL on MRSA (ATCC 33591)	[74]
Spherical AgNPs with size range of 16–18 nm	Inhibit MRSA growth at MIC value of 8 μg/mL and AgNPs cause the accumulation of ROS, which led to irreversible oxidative damage on MRSA.	[75]
Spherical AgNPs with size range of 4.5 to 26 nm	Disk diffusion assay showed an inhibition zone of 23.7 ± 0.08 mm in comparison to ampicillin treatment (26.7 ± 0.33 mm). AgNPs also exhibits MIC value of 1.2 mg/mL. ROS accumulation contributed to MRSA membrane disruption and led to cell death.	[76]

**Table 3:** Combinations of AMPs and AgNPs with addition of polymer for inhibiting *S. aureus* or MRSA growth.

AMP Type	Product Combination	Antibacterial Properties	Ref.
Nisin (antibacterial peptide produced by the *Lactococcus lactis*, which is commonly used as food preservative)	Silver-nisin nanoparticles (Ag-nisin NP)	Exhibit MIC value of 4 mg/L on MRSA in comparison to silver nitrate (16 mg/L) and nisin (4 mg/L) alone. Inhibit MRSA growth by destroying the biofilm. Ag-nisin NP showed lower cytotoxicity on human skin fibroblasts (Hs 44.Fs, ATCC^®^ CRL7024™) and human kidney epithelium cell line (HEK) compared to silver nitrate.	[87]
Daptomycin (clinically approved AMPs for medical usage)	Daptomycin-silver nanoclusters (D−AgNCs)	Complex exhibits the highest inhibitory effect against *S. aureus* in comparison to the controls (daptomycin or AgNCs alone). Inhibit growth by inducing DNA damage and ROS generation.	[88]
GL13K (amphiphilic AMPs that was developed from BPIFA2 (human salivary protein)	AgNP-dGL13K complexes (AMPs and AgNPs coated with etched Titanium (eTi) for stable nanostructure)	Exhibit excellent antibacterial properties on MRSA through in vitro and in vivo rat models.	[89]
G-Bac3.4 (amino acid sequence: CRFRLPFRRPPIRIHPP PFYPPFRPFL–NH2)	Bioconjugate G-Bac3.4 with silver nanoparticles	These bioconjugate AMPs and AgNPs exhibit antimicrobial action by internalising into MRSA and inhibiting the growth.	[90]
MBP-1 (plant antimicrobial peptide)	MBP-1 and silver nanoparticles combination	The MIC of MBP-1 is 0.6 mg/mL while MIC for silver nanoparticles were 6.25 and 12.5 mg/L. MIC of silver nanoparticles and MBP-1 combination was found to be 3.125 mg/mL and 6.25 mg/mL, respectively, on *S. aureus*. Faster wound healing can be observed on rats infected with *S. aureus*.	[37]

## Data Availability

Data sharing not applicable.
